# Loss of MafA and MafB expression promotes islet inflammation

**DOI:** 10.1038/s41598-019-45528-x

**Published:** 2019-06-24

**Authors:** Tania Singh, Jesper K. Colberg, Luis Sarmiento, Patricia Chaves, Lisbeth Hansen, Sara Bsharat, Luis R. Cataldo, Monika Dudenhöffer-Pfeifer, Malin Fex, David Bryder, Dan Holmberg, Ewa Sitnicka, Corrado Cilio, Rashmi B. Prasad, Isabella Artner

**Affiliations:** 1 Stem Cell Center, Lund University, Klinikgatan 26, Lund, 22184 Sweden; 2 Lund University Diabetes Center, Jan Waldenströms gata 35, Malmö, 21428 Sweden

**Keywords:** Gene expression, Autoimmunity

## Abstract

Maf transcription factors are critical regulators of beta-cell function. We have previously shown that reduced MafA expression in human and mouse islets is associated with a pro-inflammatory gene signature. Here, we investigate if the loss of Maf transcription factors induced autoimmune processes in the pancreas. Transcriptomics analysis showed expression of pro-inflammatory as well as immune cell marker genes. However, clusters of CD4+ T and B220+ B cells were associated primarily with adult *MafA*^−/−^*MafB*^+/−^, but not *MafA*^−/−^ islets. MafA expression was detected in the thymus, lymph nodes and bone marrow suggesting a novel role of MafA in regulating immune-cell function. Analysis of pancreatic lymph node cells showed activation of CD4+ T cells, but lack of CD8+ T cell activation which also coincided with an enrichment of naïve CD8+ T cells. Further analysis of T cell marker genes revealed a reduction of T cell receptor signaling gene expression in CD8, but not in CD4+ T cells, which was accompanied with a defect in early T cell receptor signaling in mutant CD8+ T cells. These results suggest that loss of MafA impairs both beta- and T cell function affecting the balance of peripheral immune responses against islet autoantigens, resulting in local inflammation in pancreatic islets.

## Introduction

Development of organ-specific immune disorders is caused by altered gene expression and/or function in both target cells as well as the immune system^1–3^. Such genetic disturbances generate a pro-inflammatory cellular microenvironment, which with additional defects in immune cell function may lead to disrupted self-tolerance mechanisms against autoantigens^3–5^. One such dominant impaired immunopathology is observed in type 1 diabetes (T1D), where pancreatic beta (β) cells are selectively destroyed by autoimmunity that ultimately leads to insulin deficiency^6,7^. Genome-wide association and linkage studies have identified several genetic loci associated with T1D susceptibility, however, the precise mechanisms which are perturbed by these mutations and how they initiate autoimmune reactions against islet cells have not yet been described^1,8–11^. Environmental factors such as virus infection, gut microbiota, and toxins may serve as additional exogenous triggers for initiation of T1D, and partially account for the alarming increase in global T1D incidence rate of children (<5 years) and young people (<20 years) during the last decades^12,13^.

Progression of immune-mediated tissue destruction in autoimmune diseases is mainly caused by activated T cells through their enhanced effector and cytotoxic actions. Multiple factors such as antigen concentration, T cell receptor avidity and T cell sub-type proportions (effector vs. regulatory vs. helper) are crucial for the outcome of autoimmune reactions. The TCR/CD3 complex is a fundamental unit of T cells, as TCR signaling controls thymic T cell development and selection, as well as regulates stimulation of effector functions in the periphery^14,15^. Efficient early TCR complex activation initiates a cascade of intracellular signaling network which is controlled by tyrosine kinases (LCK, FYN, ZAP70), phosphatases (PTPRC, PTPN22) and adaptor proteins (LAT, LCP2). The duration and intensity of antigen stimulation also influence TCR signaling, with additional co-stimulatory signals simultaneously enhancing an appropriate response necessary for complete activation. Thus, it is crucial that TCR signaling is tightly regulated, as abnormalities in individual signaling components may lead to disruption of central tolerance mechanisms in the thymus resulting in an accumulation of autoreactive T cells. Complete loss of function or partial defects in TCR signaling result in immunodeficiency, but autoimmune reactions have also been described^16^ as genetic alterations of TCR signaling genes like *ZAP70*, *SH2D1A*, *LAT*, and *PTPN22* are associated with arthritis, multiple sclerosis, T1D and thyroiditis^17–21^. Even though TCR signaling pathways are well defined in both mice and humans, the mechanisms by which certain genetic variations cause autoimmune dysregulation are not completely understood.

MafA and MafB transcription factors regulate pancreatic β and alpha (α) cell development^22^, maturation^23,24^ and function^25,26^. Loss of MafB also impairs macrophage self-renewal^27^ and anti-inflammatory polarization of macrophages^28^ while MafA has been reported to regulate thymic insulin expression^29^. MafA and MafB have been described as transcriptional activators^30^, but recent reports have demonstrated that MafB inhibits interferon beta transcription^31^, while MafA directly represses TNFα transcription^32^. Our recent results show that *MAFA* expression is negatively correlated with pro-inflammatory cytokine expression in human islets and restricts virus propagation in *MafA*^−/−^ islets due to an enhanced anti-viral response generated by interferons and interferon-induced genes^33^, suggesting a role for MafA in preventing adaptive autoimmune reactions against islet cells by regulating the islet inflammatory microenvironment.

Here, we show that *MafA* mutant islets have pro-inflammatory and immune cell expression signatures and that *MafA* and *MafB* compound mutant animals (*MafA*^−/−^*MafB*^+/−^) have islet-specific inflammation with an accumulation of CD4+ T and B cells, indicating an adaptive autoimmune response against islet cells. Additional *MafA* expression domains were detected in lymph nodes and bone marrow with expression being reduced in *MafA* mutant CD3+ thymocytes. Conditional deletion of MafA in hematopoietic cells also caused islet inflammation. *MafA* mutant CD4+ T cells were activated while CD8+ T cells had a reduced activation profile and expression of early TCR signaling components. Expression of *Lck*, *Fyn*, *Zap70*, *Lat* and *Ptpn22* was impaired which coincided with reduced Zap70 phosphorylation upon acute TCR stimulation. These results suggest that MafA expression in islets and immune cells is critical for preventing abnormal autoimmune reactions against islets.

## Results

### *MafA* deficient islets express pro-inflammatory chemokines and genes associated with T, B, and antigen presenting cells

**Figure 1:**
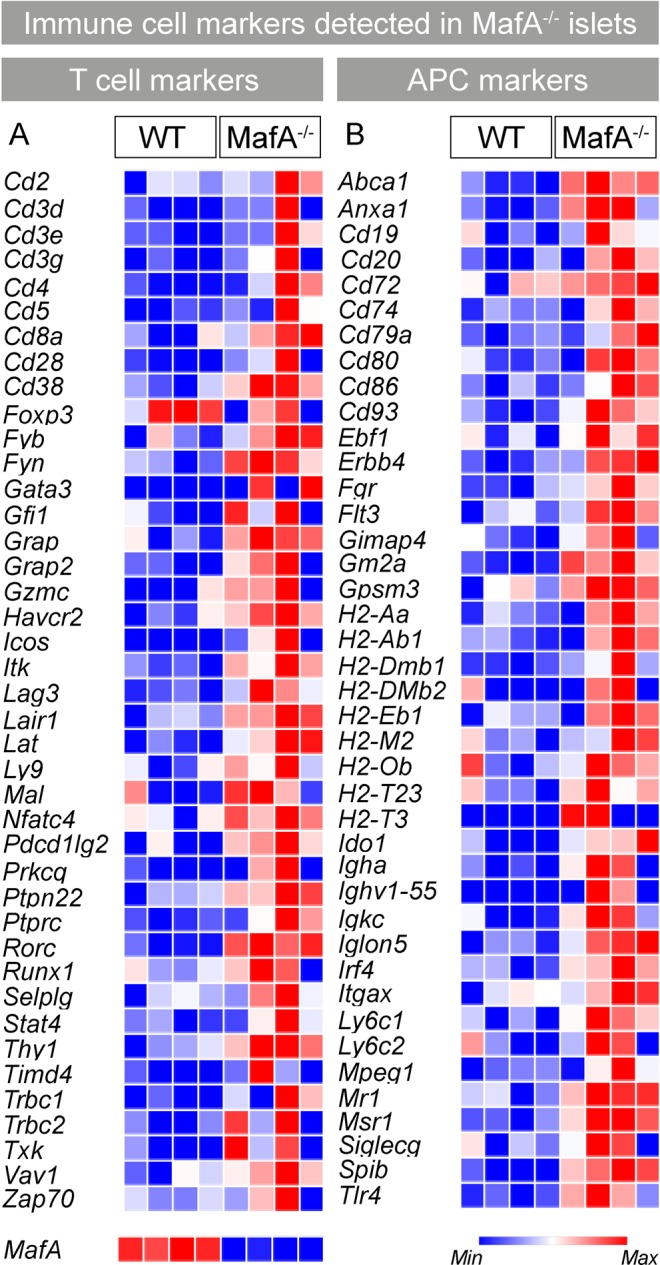
8 months old *MafA*^−/−^ islets showed enrichment of immune cell-specific genes. (**A**,**B**) Heat maps generated using Log-CPM values show (**A**) T cells and (**B**) macrophages, B and dendritic cells (APC) specific genes expression in islets. (**B**) Color gradient (last row on right heatmap) represents expression of each gene/sample (red = highest expression (Max); deep blue = lowest expression (Min); white = intermediate expression). (**A**) Last row on left heatmap shows MafA expression. N = 4 mice/genotype. Log-FC, P Value and FDR values are shown in Supplementary Table S1.

Previous studies have shown that reduced *MAFA* expression in human islets increases pro-inflammatory cytokines and induces interferon-mediated signaling pathways^33^. To determine if these global changes in gene expression were also observed upon ablation of *MafA*, RNA-sequencing (RNA-seq) of wild type^34^ and *MafA*^−/−^ islets was performed. Chemokines and chemokine receptor expression levels were elevated in *MafA*^−/−^ islets (Table 1, Supplementary Fig. S1), though a systematic enrichment of interferons was not detected. Interestingly, *MafA*^−/−^ islets had elevated expression of immune-cell specific genes (Fig. 1A,B; Supplementary Table S1 showing corresponding Log-FC, P value and FDR for each gene) indicating that islets were infiltrated/associated with both innate as well as adaptive immune cells. This included expression of T cell-enriched genes (Fig. 1A) and marker genes for B and antigen presenting cells (APC) (Fig. 1B). Expression of CD3, CD4, and CD28 was detected, while CD8 was not significantly enriched in all samples, suggesting a prevalence of CD4+ T cells. *FoxP3*, which is a marker for regulatory T cells, was not differentially expressed, suggesting that islet T cells were non-regulatory T cells. Overall, upregulation of immune cell marker gene expression was not homogenous in all mutant samples reflecting a dynamic inflammatory process and that loss of *MafA* initiates pro-inflammatory processes within the islets which may induce an adaptive immune cell response against islet cells.

**Table 1:** Chemokines and chemokine receptor expression in MafA^−/−^ islets.

Genename	Log-FC	P Value	FDR
**Chemokines**			
*Ccl8*	−5.52	0.0049	0.0347
*Ccl11*	−2.94	3.5945e-07	1.0325e-14
*Ccl17*	−4.74	0.0664	0.2403
*Ccl22*	−2.23	0.0035	0.0267
*Cx3cl1*	−0.99	6.0679e-05	0.0009
*Cxcl14*	−1.73	0.0019	0.0163
*Cxcl12*	−1.76	0.0327	0.1450
*Cxcl10*	3.24	0.0233	0.1130
**Receptors**			
*Cxcr3*	−4.00	0.0057	0.0391
*Cxcr4*	−0.93	0.0278	0.1283
*Cxcr6*	−2.77	0.0020	0.0170
*Ccr2*	−1.93	0.0265	0.1240
*Cx3cr1*	−2.03	4.0821e-05	0.0006

8 months old *MafA*^−/−^ islet RNA-seq data for chemokines and chemokine receptor genes. Log fold change (Log-FC) negative values represent upregulated genes whereas positive values represent downregulated gene in *MafA*^−/−^ islets. N = 4 mice/genotype, P value and false discovery rate (FDR) are shown.

### Altered islet morphology, islet-specific inflammation, and loss of β cell area in *Maf* deficient pancreata

**Figure 2:**
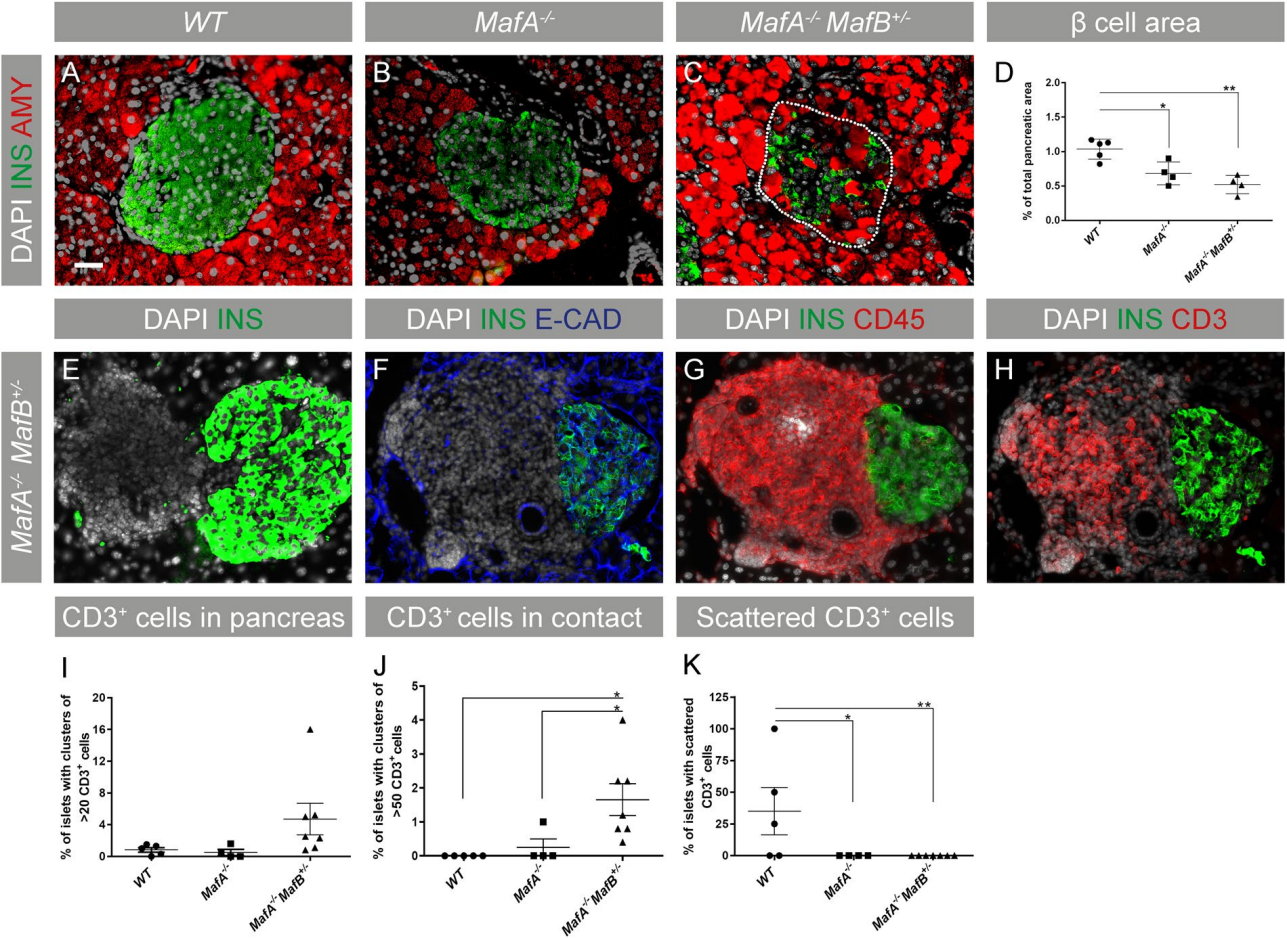
*MafA*^−/−^*MafB*^+/−^ islets have severe morphological changes and immune cell infiltration. (**A**–**C**) Histological images of 6-month-old wt and *Maf* mutant islets stained for amylase (red), insulin (green) and nucleus (DAPI; grey). (**A**,**B**) No amylase + exocrine cells were found inside the islets of wt and *MafA*^−/−^ mice. (**C**) In *MafA*^−/−^*MafB*^+/−^ islets amylase + cells were observed within the remnants of islets (dotted line indicate the border of an islet). (**D**) β cell area of wt, *MafA*^−/−^ and *MafA*^−/−^*MafB*^+/−^ is indicated in % pancreatic area. (**E**–**H**) *MafA*^−/−^*MafB*^+/−^ islets were found next to the uncharacterized condensed cluster of cells, which were negative for (**F**) epithelial marker e-Cadherin (blue) but were (**G**,**H**) positive for CD45 and CD3 markers (red); scale bar is 40 µm. (**I**) Percentage of islets with clusters of CD3+ cells (>20 cells) in wt and *Maf* deficient mice. (**J**) CD3+ cell clusters (>50 cells) in direct contact with islets were only detected in *MafA*^−/−^*MafB*^+/−^ pancreata. (**K**) CD3+ cells scattered/not in contact with islets were only observed in wt but not in *MafA*^−/−^ and *MafA*^−/−^*MafB*^+/−^ pancreata. CD3+ cells were counted in 5–7 animals per genotype. wt samples contain data points from wt and *MafB*^+/−^ samples, no inflammation was observed in this genotype. Immunohistochemical analysis was performed in at least 3 replicates. Graphs are shown with mean ± SEM and data were analyzed with one-way ANOVA Tukey’s multiple comparison test with *P value ≤ 0.05; **P value ≤ 0.01 considered as significant.

Previous studies have shown that MafA and MafB cooperate to promote β cell development and function^35^ suggesting that these genes may also synergistically prevent inflammatory processes. To assess if the loss of MafA and MafB results in altered islet morphology, reduced β cell mass, and accumulation of immune cells, 6 months old wt, *MafA*^−/−^ and *MafA*^−/−^*MafB*^+/−^ pancreata were analyzed by immunohistochemistry. Amylase-producing exocrine cells were only detected inside *MafA*^−/−^*MafB*^+/−^ islets (Fig. 2A–C) with scattered β cells present within exocrine structures (data not shown). Wt β cells constitute approximately 1% of the total pancreas area, while the β cell area of *MafA*^−/−^ and *MafA*^−/−^*MafB*^+/−^ mice was reduced by 32% and 48%, respectively (Fig. 2D). These changes in β cell area were also accompanied by impaired glucose clearance and glucose-stimulated insulin secretion, while insulin tolerance was unchanged (Supplementary Fig. S2). The observed disruption and destruction of islet structures in *MafA*^−/−^*MafB*^+/−^ pancreata were accompanied by the presence of condensed clusters of cells adjacent to islets (Fig. 2E) which did not express the epithelial protein E-cadherin (Fig. 2F), but immune cell-specific proteins CD45 (Fig. 2G) and CD3 (Fig. 2H–J). Other organs did not display any signs of T cell infiltration (Supplementary Fig. S3), suggesting that inflammatory processes were pancreas-specific. Few wt CD3+ T cells were detected (Fig. 2I) and these cells were scattered throughout the pancreatic tissue (Fig. 2K). In contrast, *MafA*^−/−^ T cells were only observed in contact with islets (Fig. 2G–J) suggesting that *MafA*^−/−^ CD3+ cells were attracted to islet cells. However, a significant accumulation of larger CD3+ T cell clusters (p = 0.0132) was only detected in *MafA*^−/−^*MafB*^+/−^ pancreas (Fig. 2I,J), suggesting that only combined loss of MafA and MafB induced large-scale accumulation of adaptive immune cells in the pancreas.

### Islet inflammation is characterized by the presence of CD4+ T and B cells in *Maf* deficient mice

**Figure 3:**
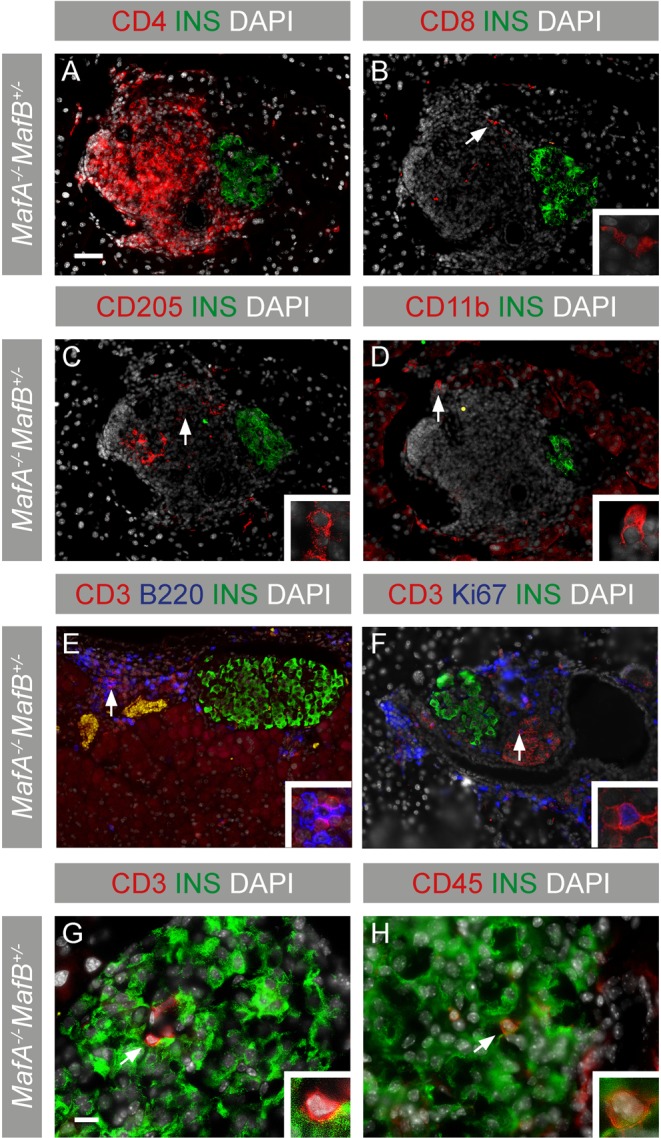
Islet inflammation in 6 months *MafA*^−/−^*MafB*^+/−^ pancreata. Inflammatory immune cells were characterized with the following markers on adjacent cryopreserved pancreatic sections: (**A–H**) Nucleus (DAPI; grey), insulin (green), (**A**) CD4 (red), (**B**) CD8 (red), (**C**) dendritic cell marker CD205 (red), (**D**) macrophage/monocyte CD11b (red), (**E–F**) T cell marker CD3 (red), B cell marker B220 (blue) and (**F**) proliferation marker Ki67 (blue). (**G**,**H**) CD3+ and CD45+ immune cells within islets. (**E–H**) Arrows point to cells magnified in inserts and scale bar is 40 µm.

To characterize the immune cells infiltrating *MafA*^−/−^*MafB*^+/−^ pancreata further, immunohistochemical analyses were performed. The majority of T cells was positive for CD4, while CD8 was only detected in a small portion of T cells (Fig. 3A,B). Few CD205+ dendritic cells and CD11b+ macrophages (Fig. 3C,D) were also detected in the immune cell clusters. Expression of the B cell marker B220 was prominent and B cells appeared to be intermingled with T cells (Fig. 3E) indicating ongoing crosstalk between antigen presenting B, T and islet cells. T cells were positive for Ki67 (Fig. 3F, 14.6% ± 3.9 s.d.) and were detected inside the islets, indicating an active and proliferating immune response against β cells (Fig. 3G,H). The presence of CD4+ T and B cells in *Maf* deficient pancreata is a characteristic feature of an autoimmune type of inflammation as observed in T1D^36^, a notion that is further supported by the absence of clusters of macrophages which are the major mediators of inflammatory processes in type 2 diabetes^37^.

### *MafA* is expressed in lymphoid organs and CD3+ T cells

**Figure 4:**
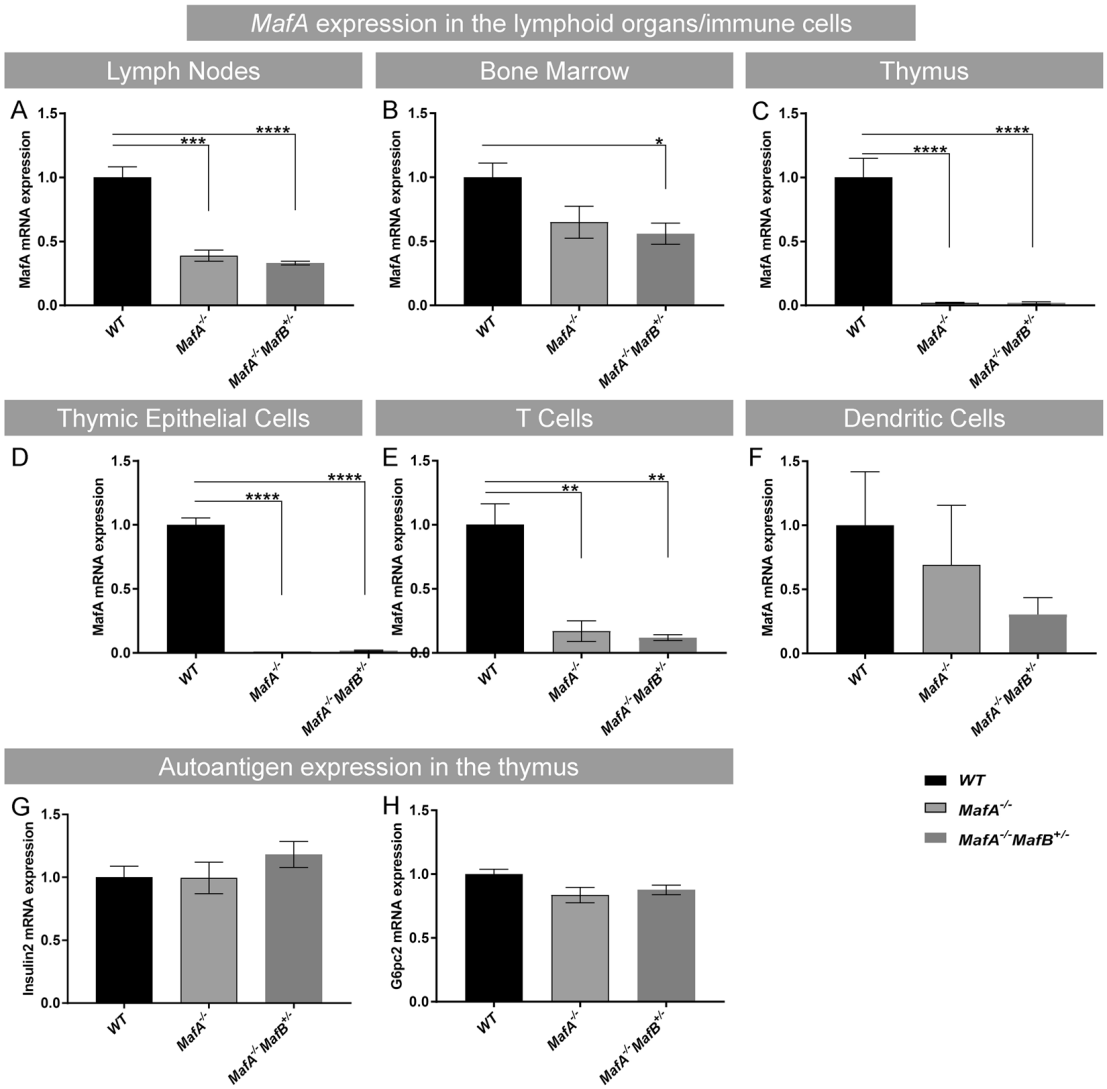
*MafA* is expressed in TEC and T cells. (**A–C**) *MafA* expression in 2 months old (**A**) lymph nodes, (**B**) bone marrow, and (**C**) in postnatal day (**P**) 7 thymus. (**D–F**) *MafA* expression was predominantly detected in sorted P0,5 (**D**) wt TEC and (**E**) wt CD3+ thymocytes with reduction observed in the *Maf* mutants whereas no clear expression and reduction was detected in (**F**) dendritic cells. Results are shown as mean ± SEM from 3–6 mice/genotype and data are represented from 3 independent experiments. (**G**,**H**) Islet autoantigens *Insulin2* and *G6pc2* expression were assessed in P7 wt and *Maf* mutant thymus. Graphs are shown as mean ± SEM with 4 mice/genotype and data are represented from 2 independent experiments. Data are analyzed with one-way ANOVA Tukey’s multiple comparison test (one way ANOVA) with *P value ≤ 0.05, **P value ≤ 0.01, ***P ≤ 0.001 and ****P ≤ 0.0001 considered significant.

**Figure 5:**
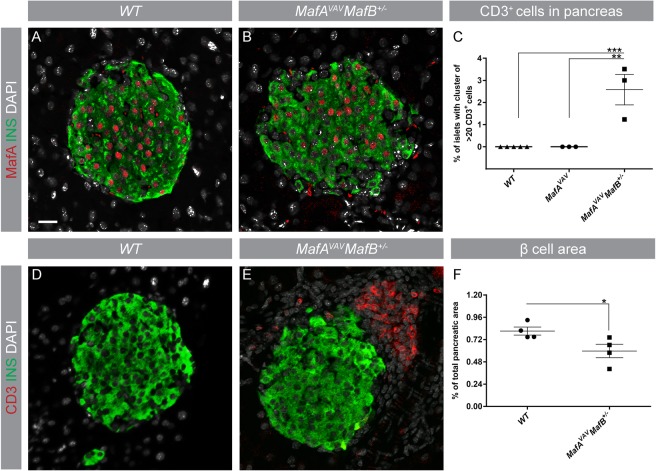
*MafA* deletion in hematopoietic (*MafA*^*VAV*^) cells causes islet inflammation and reduction of β cell area. (**A**,**B**) MafA (red) expression was assessed in (**A**) wt and (**B**) *MafA*^*VAV*^*MafB*^+/−^ islets stained with insulin (green) and DAPI for nucleus (grey). (**C**) Quantification of CD3 in *MafA*^*VAV*^*MafB*^+/−^ pancreas (**D**,**E**). CD3+ T cells (red) and insulin (green) in wt (**D**) and *MafA*^*VAV*^*MafB*^+/−^ (**E**). (**F**) Quantification of β cell area in *MafA*^*VAV*^*MafB*^+/−^ pancreas. Representative images are from at least 3 experiments; N = 3–5 mice/genotype with scale bar of 20 µm. (**E**,**F**) Graphs are shown with mean ± SEM; N = 3–5 mice/genotype and are analyzed with (**C**) Tukey’s multiple comparison test and (**F**) unpaired T test with *P value ≤ 0.05, **P ≤ 0.01, ***P ≤ 0.001 as significant.

The aberrant accumulation of immune cells adjacent to *MafA*^−/−^*MafB*^+/−^ islets may be a result of enhanced cytokine expression in islets^33^, but previous reports have even described functions of Maf transcription factors in immune cells^38–40^. To assess if MafA and MafB were present in lymphoid organs, we analyzed gene expression using quantitative gene expression analysis (Fig. 4A–F, Supplementary Fig. S4). *MafB* expression was found in lymph nodes and spleen, while *MafA* expression was detected in lymph nodes, bone marrow, and thymus (Supplementary Fig. S4), with MafA expression being significantly reduced in *MafA* mutant lymph nodes (*MafA*^−/−^ p = 0.0002; *MafA*^−/−^*MafB*^+/−^ p < 0.0001) and thymus (<0.0001) (Fig. 4A–C). At the cellular level, thymic MafA expression was significantly reduced in sorted thymic epithelial (TEC) (Fig. 4G, p < 0.0001) and T cells (Fig. 4H, respective p values were *MafA*^−/−^ p = 0.0085; *MafA*^−/−^*MafB*^+/−^ p = 0.0020) whereas dendritic cells had no significant reduction in *MafA* expression (Fig. 4I, *MafA*^−/−^ p = 0.8230; *MafA*^−/−^*MafB*^+/−^ p = 0.3581). No alterations in major islet autoantigen gene expression of *Insulin2 (*Fig. 4M) and *G6pc2* (Fig. 4N) were detected in the thymus. To evaluate if loss of MafA in immune cells contributes to the observed islet inflammation hematopoietic cell-specific (*MafA*^*VAV*^) knock-out mice were analyzed. MafA expression was present in both wt and *MafA*^*VAV*^*MafB*^+/−^ β cells (Fig. 5A,B). CD3+ cells were detected in *MafA*^*VAV*^*MafB*^+/−^, with 2.58% of *MafA*^*VAV*^*MafB*^+/−^ islets being in contact with condensed clusters of CD3+ T cells (Fig. 5C–E). This was accompanied by a significant reduction of β cell area in *MafA*^*VAV*^*MafB*^+/−^ pancreata (Fig. 5F, p = 0.0437). Islet inflammation was less pronounced in *MafA*^*VAV*^*MafB*^+/−^ animals suggesting that changes in *MafA* mutant T cells and the islet microenvironment contribute to the islet inflammation observed in *MafA*^−/−^*MafB*^+/−^ deficient mice.

### Loss of *MafA* alters peripheral T cell responsiveness and induces CD4+ T cell activation

**Figure 6:**
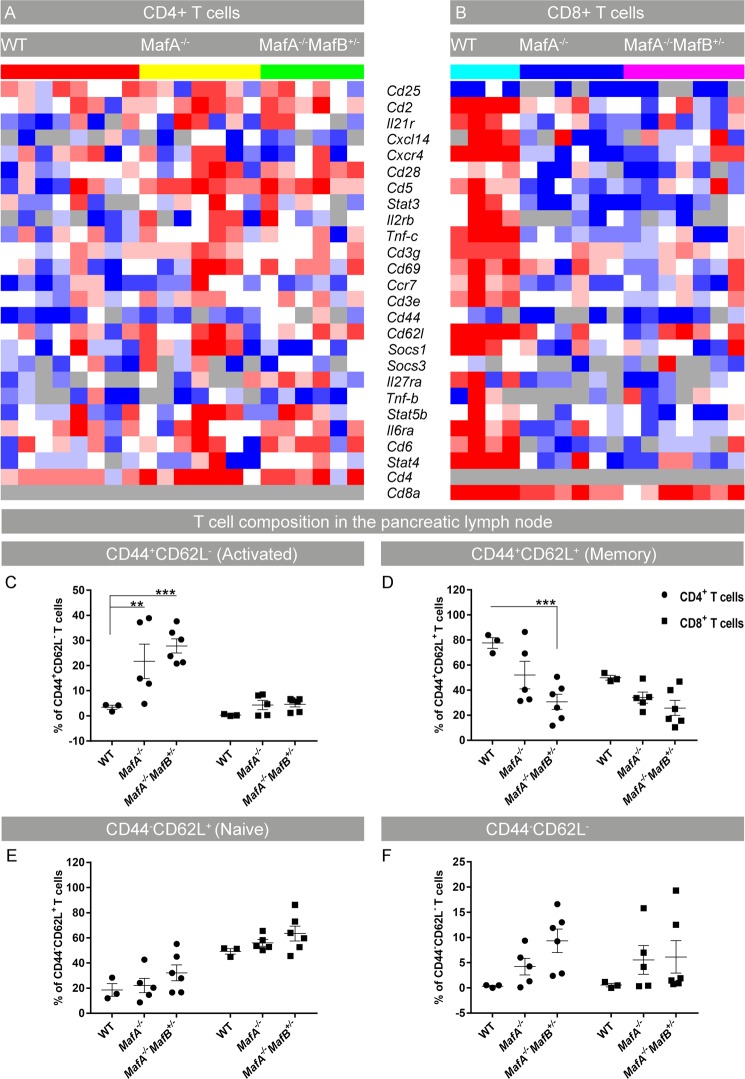
CD4+ and CD8+ T cell activation profiles in pancreatic lymph nodes from Maf deficient mice. (**A**,**B**) Heatmap showing Fluidigm gene expression from 100 pooled CD4+ and CD8+ T cells from 6–8 months-old pancreatic lymph nodes (N = 4–8). (**A**,**B**) Heatmap color gradient scheme: dark red = highest expression; dark blue = lowest expression; white = intermediate; grey = no expression. (**C–F**) Flow cytometry analysis of 6–8 months-old pancreatic lymph nodes shows percentages of CD4+ and CD8+ T cells sub-classified into activated (CD44^+^ CD62L^−^), naïve (CD44^−^CD62L^+^), memory (CD44^+^CD62L^+^) and double negative (CD44^−^CD62L^−^) states. Results are combined from 4 individual experiments with a total of 3–6 mice/genotype. (**C–F**) Graphs are mean ± SEM and analyzed with two-way ANOVA Tukey’s multiple comparison tests with **P value ≤ 0.01 and ***P ≤ 0.001.

Immunological disorders are accompanied by changes in immune cell characteristics of local draining lymph nodes due to ongoing activation, clonal expansion and peripheral tolerance processes^41–44^. To evaluate if loss of *MafA* affected these activation processes, changes in gene expression profiles of CD4+, CD8+ T cells, and APC were analyzed using a multiplexed gene expression Fludigm BioMark platform. Expression of genes characteristic for T cell activation was upregulated in mutant CD4+ T cells (Fig. 6A) while gene expression was downregulated in mutant CD8+ T cells (Fig. 6B). No significant changes in gene expression profiles of dendritic cells were observed (Supplementary Fig. S5). Increased expression of co-stimulatory molecules such as CD28, CD2, CD3, CD4 and early activation marker CD69 was observed in mutant CD4+ T cells. *MafA* deficient CD4+ T cells also had increased expression of CD6, CD44 and CD62L. The activation status of peripheral T cells was assessed by flow cytometry using surface staining of CD44 and CD62L. A significant increase in the proportion of activated (CD44 +, CD62L−) CD4+ T cells was observed in *MafA* deficient cells (*MafA*^−/−^ p = 0.0094; *MafA*^−/−^*MafB*^+/−^ p = 0.0005) whereas mutant CD8+ T cells showed no signs of activation (Fig. 6C). Interestingly, the percentage of memory CD4+ T cells was significantly reduced in *MafA*^−/−^*MafB*^+/−^ pancreatic lymph nodes (p = 0.0007) whereas the percentage of memory CD8+ T cells was unaltered (Fig. 6D). The proportion of naïve (CD44-CD62L+) CD8+ T cells was higher than CD4+ T cells which tended to increase in the mutants (Fig. 6E) whereas double negative (CD44−CD62L−) CD4+ and CD8+ T cells increased in the mutants (Fig. 6F). These findings indicated that mutant CD4+ T cells were hyper-responsive, while activation of CD8+ T cells was suppressed which was also reflected by the overabundance of CD4+ T cells in the immune cell clusters of *MafA*^−/−^*MafB*^+/−^ pancreata.

### TCR signaling is impaired in *Maf* deficient CD8+ T cells

**Figure 7:**
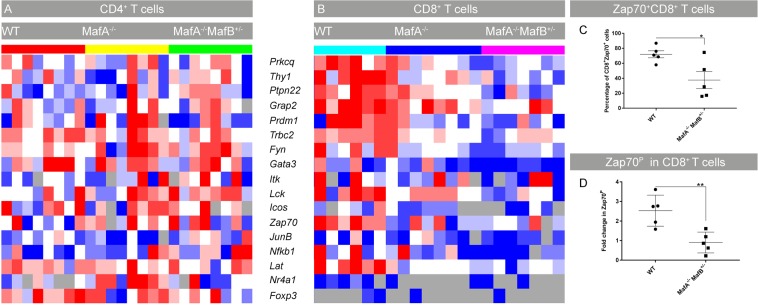
TCR signaling gene expression in CD4+ and CD8+ T cells. (**A**,**B**) Heatmap showing Fluidigm gene expression from 100 pooled CD4+ and CD8+ T cells from 6–8 months-old pancreatic lymph nodes. (**B**) Drastic reduction in the expression of TCR signaling genes was observed in *MafA*^−/−^*MafB*^+/−^ CD8+ T cells (N = 6–8 mice per genotype). Heatmap color gradient: dark red = highest expression; dark blue = lowest expression; white = intermediate; grey = no expression. (**C**,**D**) Flow cytometry analysis of wt and *MafA*^−/−^*MafB*^+/−^ CD8+ T cells showing the percentage of Zap70+ (not stimulated) and (**D**) the fold increase in Zap70 (Y319) phosphorylation in response to acute stimulation with α-CD3 and α-CD28 antibody. (N = 5 mice/genotype; 4 independent experiments). Graphs are shown as mean ± SEM and analyzed with unpaired T-test with *P value ≤ 0.05 and **P ≤ 0.01 as significant.

The lack of CD8+ T cell activation despite enhanced CD4+ T cell activation, islet inflammation, and β-cell destruction in *Maf* mutant animals led us to further investigate the mechanisms underlying T cell activation in CD4+ and CD8+ T cells. Expression of TCR signaling genes was assessed using multiplexed gene expression analysis of CD8+ T cells. Strikingly, mutant CD8+ T cells had reduced mRNA levels of genes important in the TCR signaling network (*Lck*, *Fyn*, *Lat*, *Zap70*, *Grap2*, *Prkcq*, and *Ptpn22*). These genes participate in early TCR signaling upon antigen recognition and regulate downstream T cell responses including differentiation, proliferation and cytokine production. Disruption of the TCR signaling cascade has been reported to give rise to severe immunological disorders^16^. Reduction of *Lck*, *Lat*, *Zap70* and *Prdm1* expression in T cells and a SNP mutation in *Ptpn22* have been associated with rheumatoid arthritis, systemic lupus erythematosus and T1D in humans^17,18,45–47^. Loss of expression of these genes in *MafA* deficient CD8+ T cells (Fig. 6B) but not in CD4+ T cells (Fig. 7A) indicated an abnormal T cell function through a disruption of early TCR signaling in *MafA* mutant CD8+ T cells. CD4+ T cells express the closely related cMaf transcription factor^48,49^ which may compensate for the loss of MafA, thus exhibiting comparable levels of TCR signaling genes in mutant CD4+ T cells (Supplementary Fig. S6). To assess if the loss of TCR signaling components in *MafA* mutant CD8+ T cells was directly affecting T cell activation, acute stimulation of pancreatic lymph node cells with α-CD3 and α-CD28 antibodies was performed. Flow cytometry analysis of unstimulated T cells showed a significant decrease of Zap70+CD8+ T cells in *MafA*^−/−^*MafB*^+/−^ animals (Fig. 7C, p = 0.0222) which was in line with decreased *Zap70* mRNA expression (Fig. 7B). Similarly, upon acute TCR stimulation, *Maf* deficient CD8+ T cells had a significant reduction of Zap70 phosphorylation (Y319) (Fig. 7D, p = 0.0052) confirming that early TCR signaling was impaired in *MafA*^−/−^*MafB*^+/−^ CD8+ T cells.

## Discussion

Islet inflammation is caused by the aberrant function of islet and immune cells, resulting in self-destructive adaptive immune responses against β cells. More than 60 gene loci have been associated with T1D susceptibility^8,9,50,51^, with polymorphisms mainly identified in non-coding regions of immune-regulatory genes^52^. However, genetic variants affecting function and/or expression of pancreas-specific genes may also have adverse effects on immune modulation and disease pathogenesis linked to T1D^53^. One of the major challenges following genome-wide association studies has been to decipher how disease-causing variants affect the interactions between peripheral tissues and the immune system. *MafA* polymorphisms have been linked to T1D susceptibility in the Japanese population^54^ and our recent results identified a correlation of T1D susceptibility genes with *MAFA* and *MAFB* expression in human islets^33^. Our current study shows that loss of MafA and MafB expression promotes islet inflammation, which is accompanied by hyperactivation of CD4+ and suppression of CD8+ T cell function. *MafA* mutant mice represent a genetic model of slowly developing progressive islet inflammation which resembles the inflammatory processes observed in T1D, which also result from the accumulation of T cells in the pancreas and a targeted response of T cells against islets causing β-cell destruction.

*MafA* and *MafB* are expressed in islet cells and the immune system, indicating that loss of function in these organs contributes to the inflammatory processes observed in *MafA*^−/−^*MafB*^+/−^ pancreata. Previous studies have shown that MafA suppresses pro-inflammatory cytokine expression^33^, an observation that was confirmed in our RNA-seq of *MafA*^−/−^ islets suggesting that the β cell dysfunction observed in *MafA*^−/−^*MafB*^+/−^ animals^24^ may contribute to the inflammatory processes observed. Moreover, our findings further support the notion that *MafA* function in immune cells is critical for shaping inflammatory processes against β cells.

The immune system consists of several components and cell types that jointly control tolerance mechanisms both at the central and peripheral levels. Disturbances in this immune cell crosstalk enhance the risk of developing autoimmunity, especially when combined with tissue intrinsic triggering events. Previous studies have reported that *MafA* controls transcription of islet autoantigens in TEC and polymorphisms in the *MafA* gene are linked to T1D susceptibility^29,54^. Our gene expression analysis confirmed that *MafA* was expressed in TEC, and we identified additional expression domains in T cells. However, we could not detect changes in thymic islet autoantigen expression in *MafA*^−/−^ and *MafA*^−/−^*MafB*^+/−^ cells suggesting that *MafA* is dispensable for islet autoantigen expression in TECs. The discrepancies of our findings with previous reports^29^, which described decreased thymic insulin expression, may be due to differences in mouse models and experimental time points. Moreover, conditional deletion of *MafA* in hematopoetic cells (*MafA*^*VAV*^*MafB*^+/−^) also promoted islet inflammation and reduced β-cell area suggesting that MafA may directly contribute to immune cell function. Islet inflammation was more pronounced in total *MafA*^−/−^*MafB*^+/−^ animals indicating that MafA and MafB expression in the immune system and islets are critical for preventing islet inflammation.

Immune crosstalk in draining lymph nodes of a diseased organ is essential for the development of peripheral tolerance mechanisms and if interrupted, may lead to the development of altered adaptive immune responses. This concerns especially communication between CD4+ regulatory T cells and cytotoxic CD8+ T cells, which is required to prevent destructive autoimmunity^55^. Fluidigm gene expression analysis of sorted CD4+ and CD8+ T cells showed drastic changes in activation markers between wt and *MafA* deficient T cells, suggesting an alteration in immune responses impacting peripheral T cell function. Mutant CD4+ T cells had enhanced expression of activation marker genes reflecting increased effector functions, which confirmed our observations from *MafA*^−/−^ islet RNA-seq that identified enhanced expression of chemokine receptors (*Cxcr-3*, *-4*, *-6; Ccr-2*, *Cx3cr-1*) and interleukin genes (*IL-12*, *IL-2*) suggesting the presence of type 1 helper (Th1) adaptive immune cells. Previous studies have shown that Th1 CD4+ T cells can become auto-reactive and initiate T1D development^56^. *MafA*^−/−^ islets also showed enhanced expression of *Ccl-8*, *-11*, *-17*, *-22*, *Cx3cl-1* and *Cxcl-14* chemokines, which may further promote immune cell migration into mutant islets. This was further supported by elevated *Cxcr-3* mRNA levels, as *Cxcr-3* expression in T cells has been shown to promote effector functions^57^. Additional upregulation of *Cxcr-4* and its ligand *Cxcl-12*^58,59^ further indicated the presence of an active inflammatory environment in *MafA*^−/−^ islets. A pro-inflammatory milieu within draining lymph nodes and islets was also supported by unchanged *CD25* and *Foxp3* mRNA levels in CD4+ T cells and *MafA*^−/−^ islets indicating an absence of regulatory CD4+ T cells.

*MafA* mutant CD8+ T cells did not share the enhanced activation marks observed in CD4+ T cells, most likely due to reduced co-stimulatory receptor expression which indicates impairment in CD8+ T cell responsiveness. However, complete loss of CD8+ T cell function generally causes immunodeficiencies with a central defect lying in TCR signaling^16^. Activated TCR signaling results in cytokine production and proliferation of T cells in response to pathogens, but TCR signaling is also essential for negative selection of autoreactive T cells in the thymus, thus preventing autoimmunity^60^. *MafA* mutant CD8+ T cells had a drastic reduction in TCR signaling gene expression with putative MafA binding sites present in the regulatory regions of many of these genes. Moreover, acute stimulation of mutant CD8+ T cells resulted in reduced tyrosine phosphorylation of Zap70, a critical mediator of intracellular TCR signaling. Hypomorphic Zap70 mutations cause systemic arthritis due to positive selection of autoreactive CD4+ and CD8+ T cells and loss of peripheral T cell function^60^ partially resembling our findings in *MafA*^−/−^ T cells. However, MafA mutant CD4+ T cells showed no changes in TCR signaling gene expression. This may be due to the expression of the closely related cMaf transcription factor in CD4+, but not CD8+ T cells. Previous studies have shown that large Maf transcription factors share DNA recognition sites and have similar activation potential^30^, suggesting that cMaf may compensate for the loss of MafA in CD4+ T cells.

In conclusion, we have identified *MafA* deficient animals as a novel model of monogenic islet inflammation with enhanced pro-inflammatory cytokine expression in islets and the presence of activated CD4+ T cells. Our results demonstrate that loss of MafA promotes cytokine production in islets and disturbs activation patterns of T cells. Loss of *MafA* results in reduced functionality of CD8+ T cells by loss of expression of TCR signaling genes which results in impaired responsiveness towards TCR stimulation. This suggests that MafA is critical for maintaining peripheral CD8+ T cell effector functions which are critical for immune reactions against exogenous pathogens/infections. Our results show that alterations in T cell activation status in combination with a pro-inflammatory islet microenvironment affect the balance between immunity and autoimmunity which, under normal conditions, prevents autoimmunity.

## Research Design and Methods

### Mice

Total *MafA* (*MafA*^−/−^) knock-out animals were generated by crossing *MafA*^*fl/fl*^ ^35^ with *Sox2-Cre*^61^ transgenic animals. Hematopoietic-specific *MafA* mutant mice were generated by breeding *MafA*^*fl/fl*^ with *VAV-Cre*^62^ transgenic animals. Compound mutant mice were generated by intercrosses of *MafB*^+/−^ ^63^ with *MafA*^−/−^ animals. All methods were approved by the Animal Welfare and Ethics committee in the Lund-Malmö region (Jordbruksverket; permit numbers: M 43-13, M 47-12, M 385-12). All experimental procedures were carried out in accordance with Swedish national guidelines.

### Immunohistochemistry assays

6–8 months old pancreata from wt and *Maf* mutant mice were processed for paraffin (6μm sections) and cryo-embedding (10 µm sections), respectively and immunostained as described previously^30^ with the following antibodies: guinea pig α-insulin (1:2000, Millipore/1:800, DAKO), mouse α-glucagon (1:2000, SIGMA), rabbit α-MafA (1:2000, Bethyl Laboratories), rabbit α-MafB (1:1000, Bethyl Laboratories), rabbit α-amylase (1:2000, Sigma), rabbit α-CD3 (1:500, Abcam), rat α-e-cadherin (1:400, TaKaRa), rat α-CD45 (1:100, AbD Serotec), rat α-CD4 (1:500, BD), rabbit α-CD8 (1:500, Biosite), rat α-CD205 (1:200, AbD Serotec), rat α-CD11b (1:200, AbD Serotec), rat α-CD45R/B220 (1:200, Abcam), rabbit α-CCL17 (1:100, Abcam) and mouse α-Ki67 (1:500, BD). Cy2-, Cy3- and Cy5- conjugated α-guinea pig, α-rabbit, α-mouse, α-rat secondary antibodies (Jackson Immuno Research Laboratories) were used at 1:500. Nuclear counterstaining was performed using 4′,6-diamidino-2-phenylindole (DAPI,1:6000, Invitrogen).

### Intraperitoneal glucose and insulin tolerance tests

Intraperitoneal glucose tolerance test (IPGTT) was performed on 3- and 6-months old wt and *Maf* mutant mice after overnight fasting (12 h) using an injection of 2 g glucose/kg body weight as previously described^64^. Measurements were taken at 0, 5, 15, 30, 60, and 120 minutes (min) after glucose administration. Insulin was measured from blood plasma samples of 6-months old mice using an enzyme-linked immunosorbent assay (Mercodia). For insulin tolerance tests, 6-months old mice were first sedated with a mixture of hypnorm (Vm21757/4000; Vetapharma; 25 µg/ml) and midazolam (Panpharma; 1.25 mg/ml). After taking blood glucose measurement at 0 time-point, 0.75 U/kg human insulin (Actrapid, Novo Nordisk) was injected in mice intraperitoneally. Blood glucose measurements were then taken at 15, 30, 45, and 60 minutes (min).

### Lymph node cell preparation for flow cytometry

Pancreatic lymph nodes were dissected from 6–8 months old mice, mashed through a 70 µM nylon mesh to generate a single cell suspension in RPMI-1640+ GlutaMAX, 5% FBS. Cell numbers were measured using an automated cell counter (Sysmex analyzer).

### Flow cytometry

Sorting of thymic cell subsets from P0.5 wt and *MafA* mutant littermates, 6–8 months old wt and *Maf* deficient pancreatic lymph nodes were conducted by flow cytometry. Cells were immunostained for 20 min at 4 °C with the fluorochrome-conjugated antibodies listed in Supplementary Table S2. Single cell suspensions were incubated with Fc block (BD) prior to antibody staining. Antibody concentrations were determined by titration, cells were washed and re-suspended in FACS buffer containing viability dye 7-aminoactinomycin D (7AAD, Invitrogen)/FVD, eFlour 506 (eBioscience). Cells were filtered through 30 µm filter caps (BD) and sorted for downstream RNA applications (FACS AriaII/III, BD) or analyzed (LSRII, BD).

### Islet isolation for RNA sequencing

Islets from 8 month-old wt and *MafA*^−/−^ mice were collected for RNA isolation and processed for RNA sequencing. Islet isolation was performed as previously described^65^. Thereafter, islets were picked under an inverted bright field microscope and processed for RNA extraction using the RNeasy Mini kit according to the manufacturer’s guidelines (Qiagen).

### RNA extraction for qPCR assay

RNA from P7 thymus, bone marrow, and lymph nodes were extracted using RNeasy mini kit (Qiagen) treated with RNase free DNaseI (Qiagen). RNA from sorted P0.5 TEC, T and dendritic cells was extracted using Trizol (Invitrogen), RNA carrier (AmpTec), and RNeasy mini kit (Qiagen) according to the supplier’s instructions. RNA quality was analyzed with an Agilent 2100 bioanalyzer. RNA samples with RIN (RNA integrity number) ≥7 were used for quantitative PCR (qPCR) assays.

### cDNA synthesis and qPCR analysis

Reverse transcription and qPCR have been described previously^64^. Primer sequences are listed in Supplementary Table S3. Gene expression data were normalized with delta C_T_ method against the geo-mean of internal control genes *HPRT* and *β-actin*. qPCR assays were run with at least 2 technical replicates for each sample with no template and no reverse transcriptase controls in StepOnePlus real time PCR system using fast SYBR green master mix (ThermoFisher Scientific).

### RNA sequencing and data analysis

Islet RNA was processed for cDNA library preparation with TruSeq Stranded Total RNA Library Prep Kit with Ribo-Zero Human/Mouse/Rat kit (Illumina) and sequenced using NextSeq® 500/550 High Output Kit v2 (150 cycles) (Illumina). Quality assessment was made pre- and post-sample preparation on a 2100 Bioanalyzer (Agilent). Output reads were aligned to the mouse reference genome (GRM38.75) using STAR v.2.4.1^66^. The dexseq_count python script was used by counting uniquely mapped reads in each exon^67^. Gene and exon expression normalizations were then performed using the TMM method, log-transformed and comparisons between groups were made using exactTest in edgeR^68^. Data were analyzed to yield Log-FC, FDR and P values.

### Gene expression analysis using Biomark Fluidigm dynamic arrays

8 months old lymph node residing CD4+, CD8+ T cells, and dendritic cells were sorted using flow cytometry (BD Aria III). The Biomark 96.96 dynamic array platform was chosen to perform qPCR gene expression on sorted cells (each sample represents 100 pooled cells) adapted from manufacturer’s guidelines as mentioned in their user guide (PN68000088 N1; Real-Time PCR analysis; pages:157–171). Cells were directly sorted into and lysed in buffer containing 10 mM Tris, pH8.0, 0.1 mM EDTA (TEKnova), SUPERase-In Rnase Inhibitor (Ambion), 10% NP-40 (Thermo Scientific) followed by cDNA synthesis using qScript cDNA Supermix (Quanta Biosciences). Specific target amplification (STA) was performed on cDNA using 2x TATAA preamp grandmaster mix (TATAA Biocenter) with 500 nM pooled KiCqStart SYBR Green Primers (SIGMA). Primer sequences used in this assay are available upon request. cDNA samples were then exonuclease-treated using exonuclease I enzyme and buffer (New England BioLabs). For the final qPCR step, sample mix was prepared by mixing 2X SsoFast EvaGreen Supermix with low ROX (Bio-Rad) and 20X DNA binding dye sample loading reagent (Fluidigm) with STA and exonuclease treated cDNA sample. Similarly, separate primer assay mix was prepared by mixing 2X assay loading reagent (Fluidigm), 1X DNA suspension buffer (TEKnova) and individual primer assay (100 μM). Before loading the 96.96 dynamic array IFC chip, it was primed with control line fluid in IFC controller MX (Fluidigm). Thereafter, both assay mix and sample mix were loaded into the inlets of 96.96 dynamic array IFC chip and finally run for qPCR reaction and detection on the Biomark system (Fluidigm). Data were analyzed by real-time PCR analysis software (Fluidigm) and SCExV webtool^69^.

### T cell activation assay and assessment of Zap70 (Y319) phosphorylation via flow cytometry

8 months old pancreatic lymph nodes were processed into single cells and cell numbers were assessed using Sysmex analyzer. 2 million cells were added to 96 well flat bottom plates (Nunc) pre-coated with each 5 µg/ml CD3e and CD28. Cells were allowed to bind and cross-link with antibodies for 15 minutes on ice followed by acute activation at 37 °C for 90 seconds. To terminate stimulation, cells were immediately fixed and permeabilized using a Foxp3 staining kit using their guidelines (eBioscience). Cells were immunostained for 20 minutes at 4 °C with the fluorochrome-conjugated antibodies listed supplementary information (Supplementary Table S2). Cells were recorded and analyzed by LSRII (BD) and Flow Jo software.

### Image analysis, quantification and statistical analysis

Immunofluorescence images were captured using Zeiss Axioplan 2 or Zeiss 780 confocal microscopes (Zeiss). Adobe Photoshop CC and Indesign CC were used for image processing. β cell area was determined by quantifying pancreatic and insulin-stained area throughout the pancreatic organ (108μm interval). The number of islets in each section throughout the pancreas and the total number of CD3+ cells around/in contact/scattered in islet vicinity was counted. Co-labeling of ki67 with CD3 was determined in immune cell clusters (2–7 clusters/mouse, N = 4). The data were analyzed by one-/two-way ANOVA analysis and tests were indicated in figure legends. P value ≤ 0.05 was considered statistically significant.

## Supplementary information

Supplementary Figures and Tables

## Data Availability

All data generated or analysed during this study are included in this published article (and its Supplementary Information files).
