# Supplementary material for: Loss of MafA and MafB expression promotes islet inflammation
Source: Sci Rep. 2019 Jun 24;9:9074. doi: 10.1038/s41598-019-45528-x (PMC6591483; doi:10.1038/s41598-019-45528-x)
Supplement: Supplementary file 1 — Supplementary Figures and Tables [file 41598_2019_45528_MOESM1_ESM.pdf]

Supplementary Information

Loss of MafA and MafB expression promotes islet inflammation

Tania Singh[1,2], Jesper K. Colberg[1], Luis Sarmiento[2], Patricia Chaves[1], Lisbeth Hansen[2], Sara Bsharat[1,2], Luis R. Cataldo[1,2], Monika Dudenhöffer-Pfeifer[1], Malin Fex[2], David Bryder[1], Dan Holmberg[2], Ewa Sitnicka[1], Corrado Cilio[2], Rashmi B. Prasad[2], and Isabella Artner[1,2]

[1]Stem Cell Center, Lund University, Klinikgatan 26, Lund, Sweden, 22184

[2]Lund University Diabetes Center, Jan Waldenströms gata 35, Malmö, Sweden, 21428

Address correspondence to:

Isabella Artner

Lund University
Lund Stem Cell Center
Klinikgatan 26
SE-22184 Lund
Sweden

Email: isabella.artner@med.lu.se

FAX: +46-46-222 3600

Tel: +46-(0)763138828

Running title: MafA and islet inflammation

Keywords: Autoimmunity, beta cells, CD4+, and CD8+ T cells, T cell receptor signaling

Supplementary Table 1

S1A. Table showing T cell-enriched genes from RNA-seq data of *MafA$^{-/-}$ islets*

Gene name	Log-FC	P Value	FDR
Cd2	-1.70508823616214	0.0740847822838874	0.258767154230931
Cd3d	-2.93251931402711	0.120161459210245	0.36089735819114
Cd3e	-2.68703512831097	0.0289970099262585	0.132244291957953
Cd3g	-4.13970671972263	0.00615325503057087	0.0416715670788676
Cd4	-2.80095718635265	0.00200820106955629	0.0168868750930379
Cd5	-1.9327973465763	0.0339615489909766	0.149327972347258
Cd8a	-1.4773964761892	0.190640030297428	0.483861944636714
Cd28	-4.46496331040664	0.00203203954788675	0.0170431785040734
Cd38	-1.86227527389279	2.2473895539357e-05	0.000377684453069327
Foxp3	0.989870315618054	0.222728777511686	0.537620371172104
Fyb	-0.782638408748187	0.0403185614247616	0.168768981969788
Fyn	-0.819819712570433	2.47636064719249e-05	0.000411597587892177
Gata3	-3.5094222729152	0.0841871189608388	0.283086507312301
Gfi1	-2.74587174536108	0.0250999950688089	0.11916725858321
Grap	-1.87993879141101	0.000144958442938802	0.00188636929095792
Grap2	-2.99491345889103	0.00343335793466157	0.0260900754626908
Gzmc	-1.70981349619042	0.648705270982951	0.925946907494971
Havcr2	-0.798337129668037	0.0219613572807103	0.108169087096254
Icos	-5.13792246396683	0.00778220442171402	0.0497311831585812
Itk	-3.41936882019709	3.37056074345199e-05	0.000529982772678012
Lag3	-1.13948148734395	0.00707192609343677	0.0462392036745257
Lair1	-1.0593486244082	0.00884441657151753	0.0552526777347951
Lat	-1.92882252988892	0.00101660484572092	0.00964437523992694
Ly9	-1.19054467171692	0.040763400433144	0.170160797582485
Mal	-1.90512698846173	0.0461867251658329	0.185867619995198
Nfatc4	-1.41948284913893	0.0123941935101158	0.0717291061496029
Pdcd1lg2	-2.52188802269647	0.00650207892862882	0.0435407413313809
Prkcq	-3.62477193048845	0.0789990673321956	0.271707672696487
Ptpn22	-2.49589432215302	0.000496709247415086	0.0053708645283018
Ptprc	-1.43483561855918	0.000972043614549515	0.00931497383643169
Rorc	-2.43696005545788	8.82137423945942e-32	1.1135024671931e-28
Runx1	-0.918430164994794	0.0649039379061889	0.236706640957708
Selplg	-1.14976302153821	0.0436472072254483	0.178815037030186
Stat4	-1.8600396041551	0.01029683412403	0.0623878315018895
Thy1	-2.93416592992051	4.15144756582975e-05	0.000636901013796204
Timd4	-3.39439905655042	0.0667076613312233	0.241155890709105
Trbc1	-3.22723728100456	0.0202739298237668	0.102297126254898
Trbc2	-2.89365676160862	0.00706439362851884	0.0462032491748925
Txk	-2.81716218921788	0.0225270300847138	0.110190882788974
Vav1	-0.793275538834022	0.0271752732020378	0.126160574108411
Zap70	-1.35086615602561	0.158382333932794	0.426931428317358

Supplementary Table 1 (continued)

S1B. Table showing APC enriched genes from RNA-seq data of *MafA$^{-/-}$ islets*

Gene name	Log-FC	P Value	FDR
Abca1	-1.15108220208543	1.74575781499669e-10	1.04382535038263e-08
Anxa1	-2.17658276085403	3.53812716256279e-06	7.72236188862528e-05
Cd19	-1.94352434840371	0.104995434450081	0.328867006636379
Cd20	-2.32116162375714	0.0824270036522696	0.279506962478866
Cd72	-0.741342577886079	0.0820814596717725	0.278645277932518
Cd74	-0.582806232968543	0.0311737945629248	0.139642969802592
Cd79a	-1.16367428852336	0.0244575085468176	0.116694466966032
Cd80	-1.46578127427131	0.0702733294846074	0.249812018251425
Cd86	-1.27927995139687	0.0108497635149405	0.0649413795634782
Cd93	-1.03124506037176	1.42424822300945e-05	0.000256421108359728
Ebf1	-1.03071901772103	0.0326308613158196	0.14480582030405
Erbb4	-2.93360846927413	0.000124948859822673	0.00165344382296503
Fgr	-2.83829171676517	0.000674963802942008	0.00685930118545838
Flt3	-2.92846357184423	0.00161776094185908	0.0141482472517245
Gimap4	-1.3587523168387	0.016445007005263	0.0878210895098391
Gm2a	-0.922782017839039	4.31607723071188e-09	1.96918856945792e-07
Gpsm3	-1.29097535355812	0.00877435620808873	0.0549055762610807
H2-Aa	-0.786025442836797	0.0223014440028309	0.109369978240518
H2-Ab1	-0.945847170381391	0.0145115065248942	0.0809590228623163
H2-DMb1	-1.7645386096654	0.00467436350398193	0.0334824127282388
H2-DMb2	-1.55861451744983	0.482214735930236	0.818817955962551
H2-Eb1	-0.680476854893068	0.0489082828202535	0.193285722635408
H2-M2	-1.57914168540092	0.0252931780546723	0.119801187946677
H2-Ob	-1.01737627066361	0.282593324647763	0.605678985880749
H2-T23	-0.767456772056539	0.00502410061655278	0.0354952083671318
H2-T3	-2.57600664864456	0.516576604578652	0.830301148318587
Ido1	-2.50509838425149	0.00811718697549412	0.0515025426613242
Igha	-6.26514324612212	0.000705004529641392	0.00712562629803473
Ighv1-55	-6.57703529630992	0.00399902095043656	0.0295005698100224
Igkc	-4.38298236742751	0.00498911340972097	0.0352809355064644
Iglon5	-1.3434881734347	0.0015018794684574	0.0133193612032867
Irf4	-2.34755493885876	0.00093076496546519	0.00898381936292888
Itgax	-1.37882190125498	0.00987757330588579	0.0603139863163212
Ly6c1	-2.07909663416729	0.000466084176985141	0.00510602631884252
Ly6c2	-1.30750364968438	0.422251463869175	0.760943489099899
Mpeg1	-0.526662017869999	0.0556017025497508	0.211354472337199
Mr1	-0.899861309086368	0.000356364044512551	0.00405455556102638
Msr1	-1.14365608823778	9.22724248695089e-06	0.000178275660328241
Siglecg	-1.61034762033193	0.181016097174908	0.466522997267591
Spib	-4.17024321395635	4.75658381743053e-06	9.99762635669187e-05
Tlr4	-1.03600002028736	0.00253848687887467	0.0202948096179353

Supplementary Table S1. **RNA-seq data showed enrichment of immune cell-specific genes in 8 months old *MafA*$^{-/-}$ islets.** (S1A, B) Shows Log-FC values which if negative represent upregulated genes in *MafA*$^{-/-}$ islets. N=4 mice /genotype, P value and FDR are shown. Log-CPM data from the same gene set is shown as heat maps in Fig 1A, B.

Supplementary Table 2

Table S2: Antibodies used for flow cytometry experiments

	Target Antigen	Conjugate	Clone	Isotype	Source
A.	For sorting P 0,5 thymic cell subsets (epithelial, dendritic and T cells)				
	α-CD45	APC	30-F11	Rat (LOU) IgG2b, κ	BD
	α-CD3e	APC-Cy7	145-2C11	Armenian Hamster IgG1, κ	BD
	α-CD326	PE-Cy7	G8.8	Rat IgG2a, κ	Biolegend
	α-CD11c	Fitc	HL3	Armenian Hamster IgG1, λ2	BD
	α-MHC II	PB	M5/114.15.2	Rat IgG2b, κ	Biolegend
	α-CD11b	AF700	M1/70	Rat (DA) IgG2b, κ	BD
B.	For pancreatic lymph node T cell activation analysis at 6-8 months of age				
	α-CD3e	BV421	145-2C11	Armenian Hamster IgG1, κ	BD
	α-CD4	BV650	GK1.5	Rat (LEW) IgG2b, κ	BD
	α-CD8a	APC	53-6.7	Rat (LOU) IgG2a, κ	BD
	α-CD44	BV510	IM7	Rat IgG2b, κ	BD
	α-CD62L	PE	MEL-14	Rat (F344) IgG2a, κ	BD
C.	For sorting 100 immune cells from 6-8 months old pancreatic lymph node (CD4+, CD8+ T, dendritic cells and macrophages)				
	α-CD45	PE-Cy7	30-F11	Rat IgG2b, κ	BD
	α-CD11c	Fitc	HL3	Armenian Hamster IgG1, λ2	BD
	α-CD11b	BV711	M1/70	Rat IgG2b, κ	Biolegend
	α-CD64	BV421	X54-5/7.1	Rat IgG1, κ	Biolegend
	α-CD24	PE	30-F1	Rat IgG2c, κ	Biolegend
	α-MHC II	APC	M5/114.15.2	Rat IgG2b, κ	Biolegend
	α-CD8a	PerCP/Cy5.5	53-6.7	Rat IgG2a, κ	Biolegend
	α-CD4	BV650	GK1.5	Rat (LEW) IgG2b, κ	BD

Supplementary Table 2 (continued)

	Target Antigen	Conjugate	Clone	Isotype	Source
D.	TCR activation assay at 8 months of age				
	α-CD3	none	145-2C11	Armenian Hamster IgG1, κ	BD
	α-CD28	none	37.51	Syrian Hamster IgG2, λ1	BD
	α-CD4	APC-Cy7	V4	Rat IgG2b, κ	Biolegend
	α-CD8a	PerCP/Cy5.5	53-6.7	Rat IgG2a, κ	Biolegend
	α-Phospho-Zap70 (Y319)	AF-647	65E4	Rabbit IgG	Cell Signaling
	α-Zap70	PE	D1C10E	Rabbit IgG	Cell Signaling

Supplementary Table S2. FACS antibody panel. (A-D) All fluorochrome-conjugated antibodies used in different flow cytometry experiments are listed in sub-sections corresponding to individually designed experiments shown in figure 4-6.

Supplementary Table 3

Table S3: Gene primer sequences used in quantitative PCR reactions

Gene name	Forward primer	Reverse primer
HPRT	5'-AGCCCCAAAATGGTTAAGGT-3'	5'-CAAGGGCATATCCAACAACA-3'
Actinβ	5'-GCTTCTTTGCAGCTCCTTCGTTG-3'	5'-TTTGCACATGCCGGAGCCGTTGT-3'
MafA	5'-GAGGAGGTCATCCGACTGAAA-3'	5'-GCACTTCTCGCTCTCCAGAAT-3'
Insulin2	5'-GGCTTCTTCTACACACCCAT-3'	5'-CCAAGGTCTGAAGGTCACCT-3'
G6pc2	5'-AGGTGACCCTAAGCCGGAC-3'	5'-TCTTTGGGTAGAAGACCATCCC-3'

Supplementary Table S3. Quantitative PCR primer sequences. Primer sequences of all the genes used in the quantitative PCR experiments shown in Figure 4.

Supplementary Figure 1

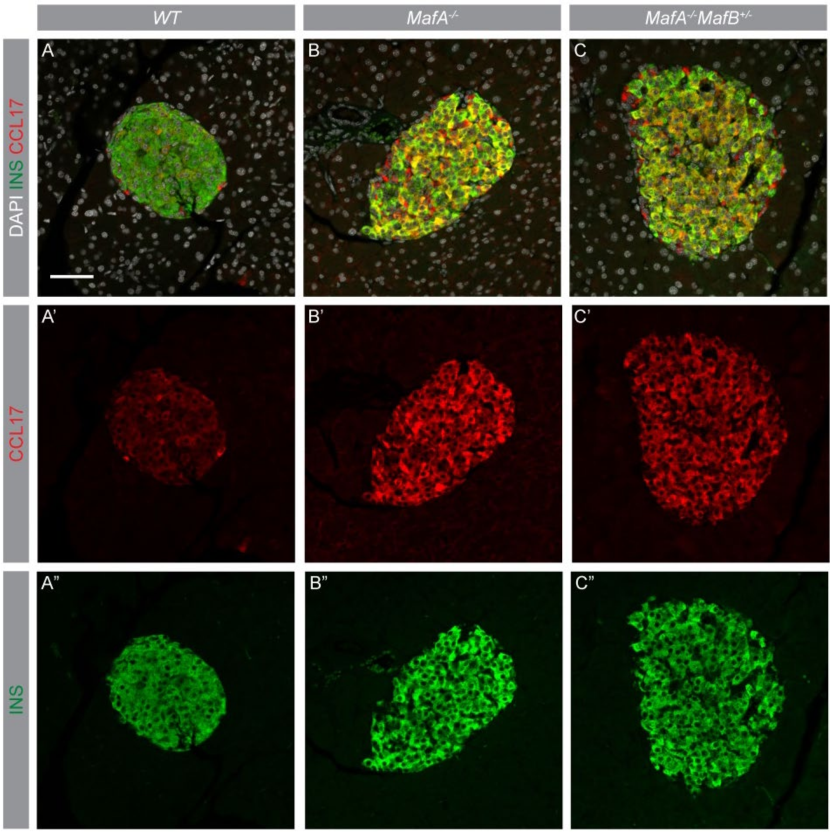

Figure S1. Ccl17 protein expression is enhanced in 6 months old *MafA*$^{-/-}$ and *MafA*$^{-/-}$*MafB*$^{+/-}$ islets. (A-C) Ccl17 (red), insulin (green) and nucleus with DAPI (grey) stainings were performed in wt (A, A', A"), *MafA*$^{-/-}$ (B, B', B") and *MafA*$^{-/-}$*MafB*$^{+/-}$ (C, C', C") pancreatic sections (scale bar is 50 µm).

Supplementary Figure 2

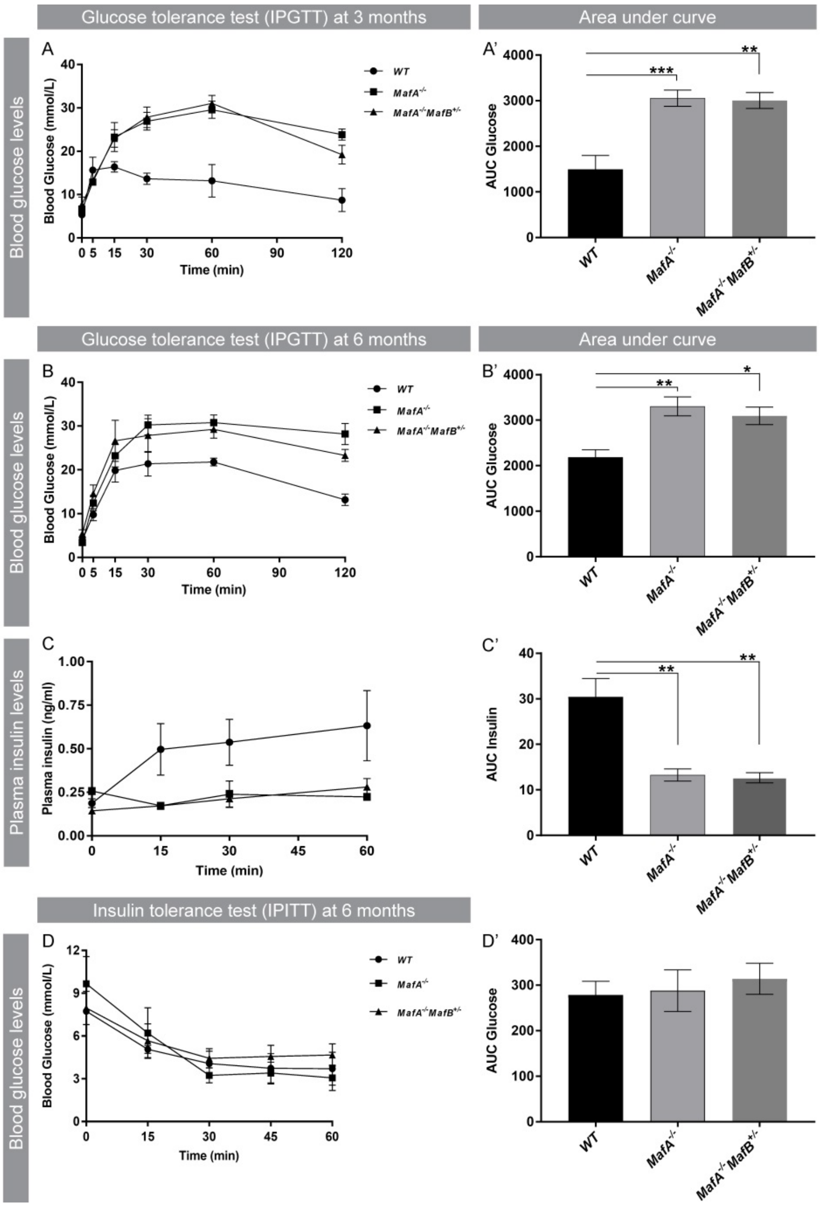

Figure S2. Maf mutant animals are glucose intolerant. (A,B) Decreased glucose clearance was observed in *Maf* mutant animals as indicated by intraperitoneal glucose tolerance test (IPGTT) (A,B) and area under the curve (AUC) (A',B') of 3 and 6 months old mice. (C) Corresponding plasma insulin levels upon glucose administration in 6 months old mice. (D) Glucose levels after insulin tolerance test in 6 months old mice. Data are represented from 6 individual experiments and are shown as mean ±SEM from at least 3 animals per genotype and were analyzed with one-way ANOVA with Tukey's multiple comparison tests with *P value ≤ 0.05, **P ≤ 0.01 and ***P ≤ 0.001 as significant.

Supplementary Figure 3

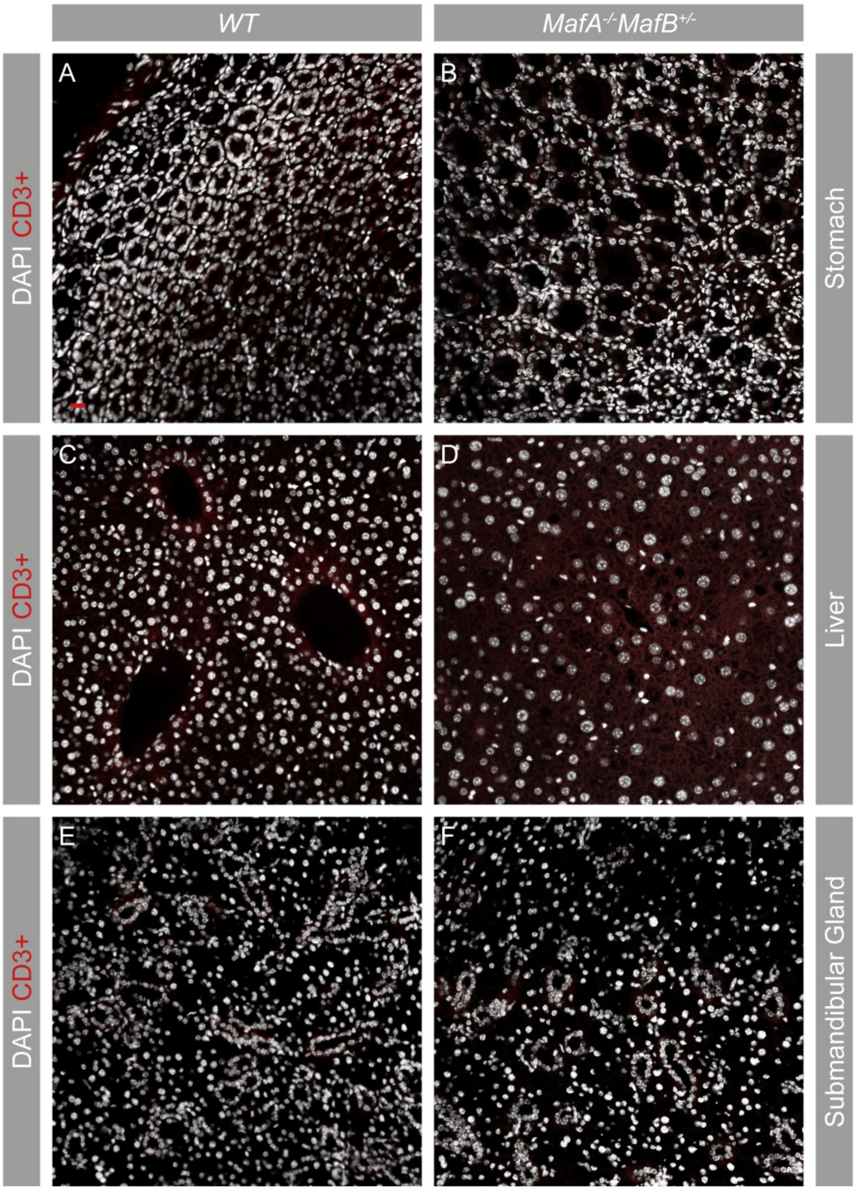

Figure S3. CD3+ T cells do not infiltrate other *MafA$^{-/-}$MafB$^{+/-}$* organs. (A-F) CD3 (red) and Nuclear DAPI (grey) stainings performed on the indicated organs (at 108 μm interval apart). No CD3+ immune cells were detected in the (A-B) stomach, (C-D) liver and (E-F) submandibular glands of wt and *MafA$^{-/-}$MafB$^{+/-}$* mice. Images are a representative of 3 mice/genotype and 6 experiments with scale bar (red) is 20 μm.

Supplementary Figure 4

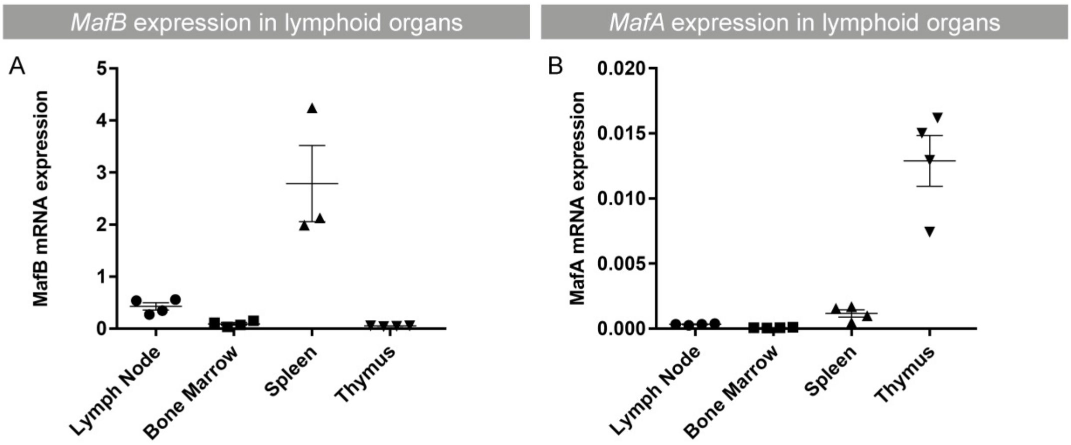

Figure S4. MafB and MafA expression in 2 months old wt lymphoid organs. (A) MafB and (B) MafA gene expression in various lymphoid organs. Graphs are shown with mean ±SEM; N=3-4 mice/genotype.

Supplementary Figure 5

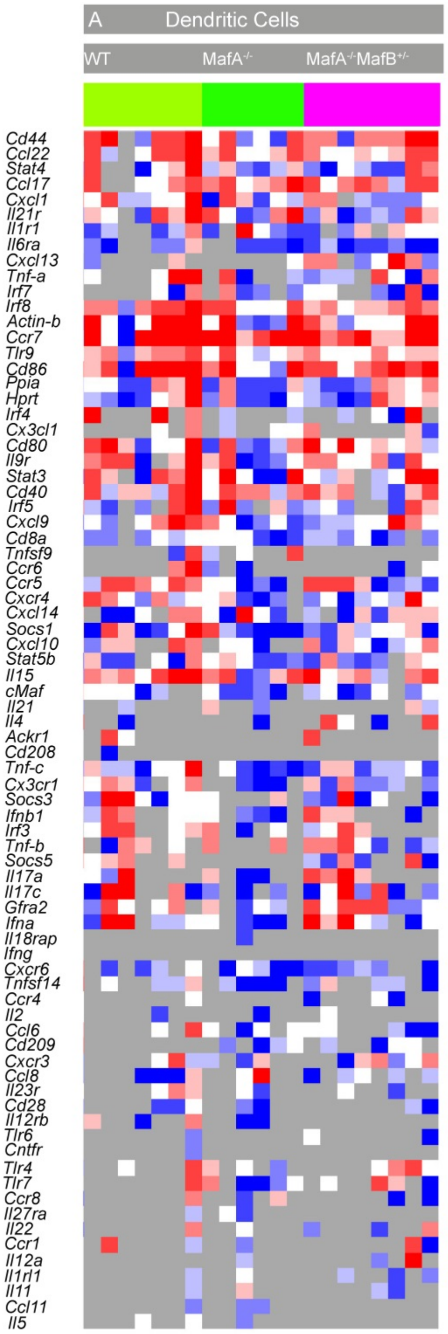

Figure S5. No changes in the activation status of dendritic cells were observed between wt and *Maf* deficient mice. (A-B) Fluidigm gene expression analysis of 100 pooled dendritic cells from 6-8 months old pancreatic lymph nodes (N=6-8/genotype). (A-B) Heatmap color gradient scheme: dark red=highest expression; dark blue=lowest expression; white=intermediate; grey=no expression.

Supplementary Figure 6

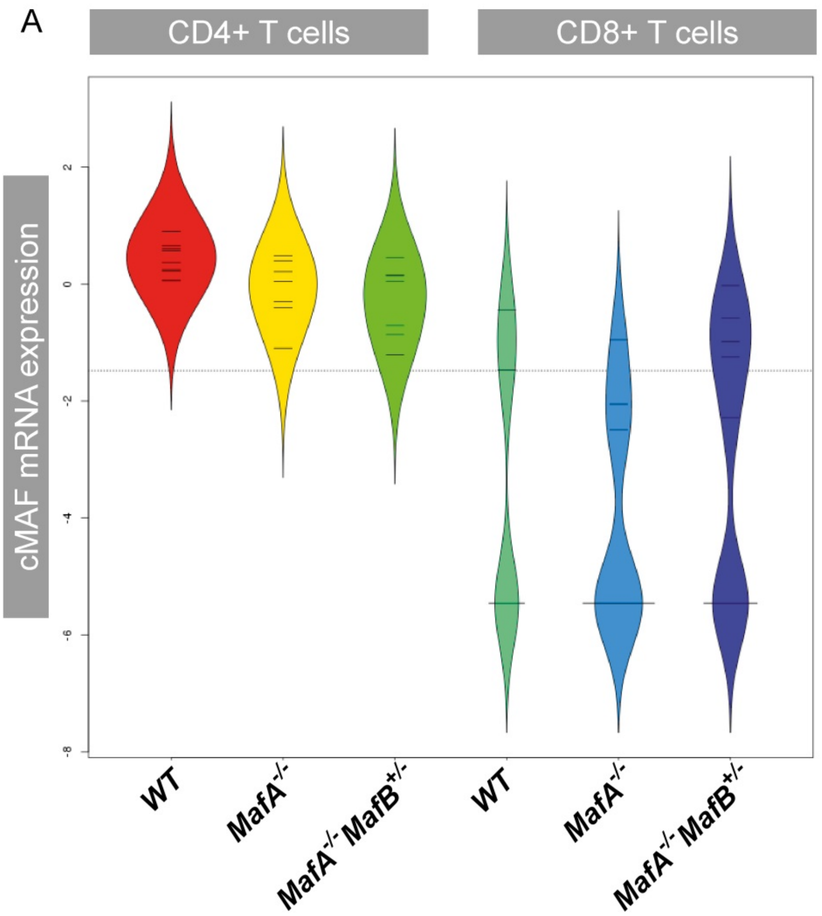

Figure S6. CD4 + T cells express cMaf. (A) cMaf mRNA expression (fluidigm gene expression analysis) in CD4+ and CD8+ T cells (N=4-8/genotype).
